# Pre-exposure prophylaxis for men and transgender women who have sex with men in Brazil: opportunities and challenges

**DOI:** 10.7448/IAS.18.4.20010

**Published:** 2015-07-20

**Authors:** Valdilea G Veloso, Fabio Mesquita, Beatriz Grinsztejn

**Affiliations:** 1 Instituto Nacional de Infectologia Evandro Chagas, Fundação Oswaldo Cruz, Rio de Janeiro, Brasil; 2 Departamento de Doenças Sexualmente Transmissíveis, AIDS e Hepatites Virais do Ministério da Saúde, Brasília, Brasil

**Keywords:** pre-exposure prophylaxis, MSM, TGW, prevention, Brazil, resource-limited setting, health system

## Abstract

**Introduction:**

The World Health Organization recently released guidelines on the use of pre-exposure prophylaxis (PrEP) for prevention of HIV infection among men and transgender women (TGW) who have sex with men based on results of randomized clinical trials. The aim of this commentary is to discuss the opportunities and challenges of incorporating PrEP into the Brazilian continuum of HIV care and prevention for men who have sex with men (MSM) and TGW.

**Discussion:**

Key aspects of the AIDS epidemic among MSM and TGW in Brazil and the comprehensive Brazilian response to the epidemic are presented. The universal access to health care provided through the Brazilian Unified Health System (SUS) and the range of prevention and care services already available countrywide to HIV-positive individuals and at-risk MSM and TGW are identified as the main facilitators for the implementation of PrEP. Limited PrEP awareness among MSM, TGW and health care providers, low HIV testing frequency and low HIV risk perception among MSM and TGW represent the core challenges to be addressed. Data generated by demonstration projects in Brazil will provide an important contribution to PrEP rollout in Brazil.

**Conclusions:**

The implementation of PrEP in Brazil is feasible. A synergistic rollout of treatment as prevention and PrEP will maximize public health and individual benefits of the country's comprehensive response to the AIDS epidemic.

## Introduction

Over the past five years, remarkable progress has been made in the fight against the HIV/AIDS epidemic. Data from randomized clinical trials demonstrating the efficacy and safety of antiretroviral drugs for the prevention of HIV acquisition [1–5] have inspired a renewed sense of optimism that the end of the AIDS era is an attainable goal [6].

Antiretroviral pre-exposure prophylaxis (PrEP), with either daily oral tenofovir disoproxil fumarate (TDF) or daily TDF in combination with emtricitabine, has been shown to be efficacious for HIV-1 prevention for high-risk men who have sex with men (MSM) and transgender women (TGW), heterosexual men and women, discordant heterosexual couples and intravenous drug users [1–4]. Data from the Pre-Exposure Prophylaxis Initiative (iPrEx) study demonstrated that oral PrEP using daily emtricitabine/tenofovir (Truvada^®^) successfully reduced the risk of HIV acquisition among MSM and TGW [1]. Protection was estimated to be over 90% in those with detectable levels of the drug in their blood, with pharmacokinetic modelling suggesting that efficacy reaches 99 and 96% with dosing of seven and four days per week, respectively [7]. Further results from the iPrEX open label extension (iPrEX OLE) reassured that this strategy can be safe and effective, and is well accepted by this population [8].

More recently, two European MSM and TGW oral PrEP clinical trials (IPERGAY and PROUD) halted their randomization phase due to the superior effectiveness of PrEP. Both studies showed 86% effectiveness of Truvada [9, 10]. The IPERGAY's results were of particular interest because this study was designed to evaluate an intermittent PrEP regimen using Truvada (on-demand), with its usage triggered by sexual activity. Currently, the use of PrEP is endorsed by the World Health Organization (WHO), the Centres for Disease Control and Prevention (CDC) and IAS–USA Guidelines [11–13].

The aim of this commentary is to discuss the opportunities and challenges of incorporating PrEP into the continuum of HIV care and prevention for MSM and TGW in Brazil.

## Discussion

### MSM and TGW – the most affected populations in Brazil

Most countries in Latin America have been affected by concentrated HIV/AIDS epidemics, and HIV infection rates in this region have changed little in the past decade, with most of the new HIV cases occurring among MSM [14, 15].

**Figure 1:**
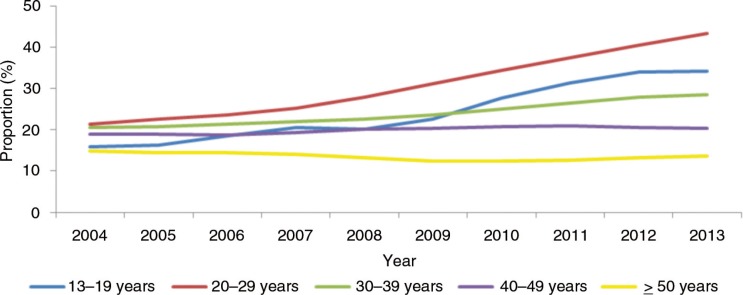
Proportion of all AIDS cases occurring in young men who have sex with men according to age in Brazil from 2004 to 2013. Source: Brazilian Ministry of Health. Department of STDs, AIDS and Viral Hepatitis [16].

The largest population of HIV-1-positive people in Latin America lives in Brazil. As of 2014, the Ministry of Health (MoH) had registered 757,042 cases of AIDS. The number of people living with HIV/AIDS in the country was estimated to reach 734,000 in 2014. However, it is the young MSM who account for nearly 40% of AIDS cases. Increases of 41.3% (aged 15–19 years) and 25.1% (aged 20–24 years) were observed from 2004 to 2013 [16] (Figure 1).

Although Brazil has an overall HIV prevalence of roughly 0.6% in the general population (0.4% among women and 0.8% among men) [17], the prevalence among MSM is 14.2% [18], which is three times higher than estimates for female sex workers, double the 5.9% estimated prevalence for drug users and 13 times higher than that for heterosexual men [17]. The prevalence of HIV infection for very young MSM (aged 15–19 years) is 4% (95% CI 1–9%) [19]. Data from three voluntary counselling and testing sites in Rio de Janeiro showed 24.8% (95% CI 19.9–29.7) prevalence among MSM, and conservatively estimated incidence among MSM to be 8.55% per year (95% CI 4.36–12.74) [20].

Although TGW represent a smaller population than MSM, they have extremely elevated HIV infection rates. A meta-analysis across 15 countries (10 were low- and middle-income countries, 5 of which were in Latin America and the Caribbean) estimated an HIV prevalence of 17.7% (95% CI 15.6–19.8) in this population, with an odds ratio of 50.0 (95% CI 26.5–94.3) for HIV infection among TGW versus all adults of reproductive age in low- and middle-income countries [21]. In Brazil, as in other Latin American countries, risks associated with HIV infection among TGW are mainly linked to high rates of sex work, limited formal education, social exclusion and violence. These factors jointly contribute to increased vulnerability and impaired access to care and prevention [22].

Studies in Brazil have shown that unprotected anal intercourse is a frequently reported sexual practice among Brazilian MSM [18, 23]. Nevertheless, a high proportion of MSM classified their risk of acquiring HIV infection as low or did not know how to rate their risk [24]. The HIV epidemic among MSM and TGW in our setting is unabated in these populations with many individuals remaining unaware either of their HIV status and the beneficial services available to them or of effective prevention strategies [15].

### Where does PrEP fit in the Brazilian continuum of HIV prevention and care?

Since the early 1990s, Brazil has implemented a comprehensive HIV prevention and care programme. Built within the Unified Health System (SUS) that provides universal health care to the entire population at no cost at the point of delivery [25], the programme includes voluntary counselling and testing services, combination antiretroviral therapy (cART), viral load and CD4 monitoring and HIV genotyping [19, 26–28]. Condom and lubricant, non-occupational post-exposure prophylaxis (nPEP), treatment for sexually transmitted infections using the syndromic approach, and hepatitis B diagnosis and treatment are also available as part of integral care. This makes it one of the most comprehensive HIV treatment initiatives implemented in a middle-income country [16, 19, 26].

Since December 2013, the Brazilian MoH has adopted the Test & Treat strategy that allows cART to be initiated promptly after HIV diagnosis, regardless of CD4 count, if the patient is willing to be treated [27]. As of December 2014, approximately 400,000 patients were receiving cART in 724 specialized care services established in the country [19].

**Figure 2:**
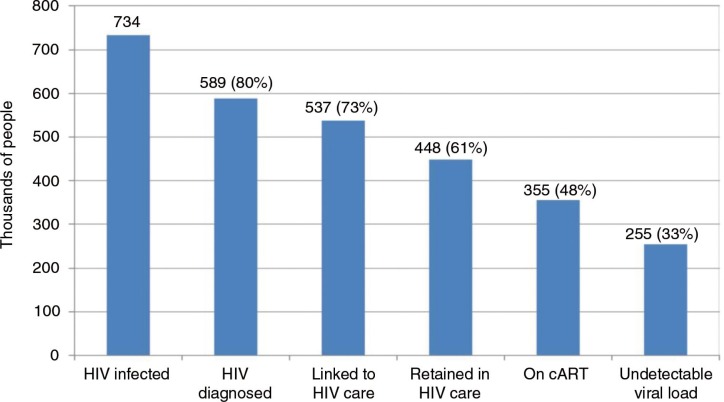
The cascade of HIV care in Brazil in 2013. Source: Brazilian Ministry of Health. Department of STDs, AIDS and Viral Hepatitis [16].

A high coverage of cART, especially within MSM networks and in the community, is crucial for reducing the spread of the epidemic among MSM. However, the cascade of care in Brazil shows that in 2013, at each level, important percentages of those living with HIV fall out of the care continuum [16]. Of the estimated 734,000 HIV- positive individuals, only 255,000 (33%) achieved an undetectable viral load [16] (Figure 2). Similar results were found when a MSM care cascade was evaluated in Rio de Janeiro, one of the epicentres of the HIV epidemic in Brazil [29].

**Figure 3:**
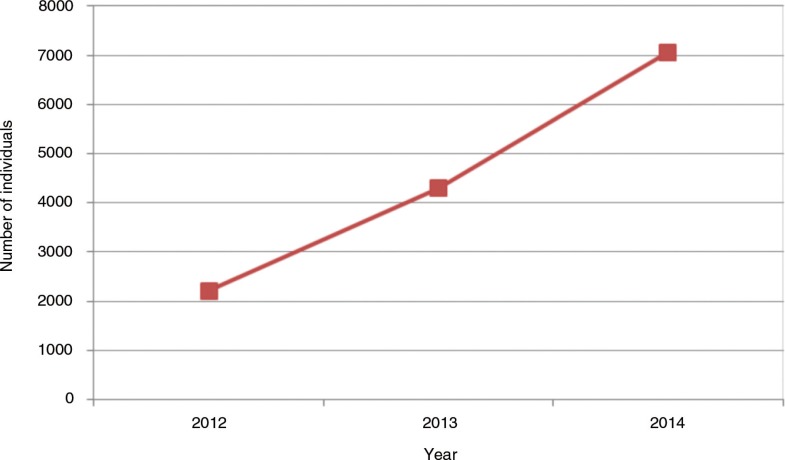
Number of individuals receiving non-occupational post-exposure prophylaxis (nPEP) from 2012 to 2014. Source: Brazilian Ministry of Health. Department of STDs, AIDS and Viral Hepatitis [16].

In this context, other prevention strategies, such as nPEP and PrEP targeting HIV-negative high-risk MSM and TGW, could play a critical role in preventing new infections. Since 2010, nPEP has been made available through the SUS [30] and its uptake has been steadily increasing (Figure 3). The addition of PrEP can further contribute to avoiding new infections among these populations and contribute to controlling the HIV epidemic in Brazil. We foresee that PrEP rollout in Brazil will take advantage of the countrywide infrastructure already established. However, critical challenges will have to be addressed.

Significant efforts are needed to increase HIV serostatus awareness and testing frequency among MSM and TGW. In 2010, 54% of MSM participating in the Brazilian National HIV Behavioral Surveillance Study reported having been HIV tested at least once in their life. However, only 19% reported an HIV test in the previous 12 months [19]. Other studies confirmed this finding, showing that less than half of the MSM enrolled had ever been tested for HIV [18, 23], and that of all the men who tested HIV positive, only half were aware of their serostatus [18, 23, 24].

Testing modalities offered in settings outside the traditional health services are critical for increasing access to HIV diagnosis among MSM and TGW. In this regard, building on a long history of partnership with civil society [31, 32], the Brazilian MoH in 2014 launched the initiative, “Viva Melhor Sabendo” (“Living better knowing”), which expanded HIV testing using oral fluid to non-governmental organizations [19]. In addition, mobile HIV testing units were provided to each one of the 27 brazilian federative units to outreach populations that may face barriers to accessing HIV testing in the context of traditional health services. In 2011, among 629 MSM surveyed though the Internet, 47% indicated a preference for home-based testing among several testing options, and up to 90% reported that they would use self-test kits to make choices about unprotected sex with regular and new partners [33]. A menu-based approach that offers different testing modalities, including novel testing strategies, such as self-testing coupled with already available testing options, might support the development of a tailored testing plan for MSM and TGW engaging in PrEP programmes in Brazil.

In the context of PrEP programmes, it is critical to make sure that individuals starting PrEP are HIV negative and remain negative while using it. In case a breakthrough HIV infection occurs, the two drug regimen used in PrEP will not fully suppress HIV replication and may select for resistance. Thus, if acute infection is suspected, PrEP initiation should be delayed until the serostatus is defined to avoid the risk of drug resistance development.

PrEP programmes will have to be coupled with HIV risk management counselling and HIV testing services, where individuals at risk are linked and can have access to PrEP and other prevention options in a stigma-free setting. Within these settings, tools to support risk assessments and adherence are crucial, as is the availability of adequate support for PrEP discontinuation.

### PrEP awareness and willingness in the MSM and TGW community

Understanding awareness and willingness to use PrEP is essential for informing public policy formulation. Evidence suggests that the concept of PrEP is well accepted by MSM; however, it is likely that there are various factors affecting PrEP uptake and adherence that may differ across countries. Surveys on PrEP in the United States, India, South Africa, Thailand, China and Peru showed that 44–92% of MSM were receptive to taking PrEP [34–37].

Awareness and willingness to use PrEP is increasing in Brazil. In 2011, only 22% of 552 MSM who participated in a self-administered web survey using Facebook had heard about the iPrEX study results. However, after a brief explanation about iPrEx and its results, 67.5% said that they were extremely likely or very likely to use daily PrEP [38]. Preliminary results from a study that is being conducted in Rio de Janeiro and São Paulo assessing awareness and willingness among MSM and TGW showed that among 734 men who reported having sex with men within 12 months and were seeking HIV testing, 60% were aware of PrEP and nearly 95% (*n*=695) demonstrated willingness to use PrEP to prevent HIV. Older age, having a steady partner and prior history of HIV testing increased the odds of PrEP awareness [39].

There is very limited data on PrEP awareness and willingness among TGW communities. In a study conducted in Thailand, acceptability of PrEP, defined as individuals who reported being “very likely” to use PrEP, was similar in MSM and transgender groups (around 40%). Correlates of PrEP acceptability among TGW were prior PrEP awareness and having private insurance, suggesting that efforts to increase awareness and accuracy of PrEP understanding and minimizing confusion of PrEP with nPEP and other biomedical HIV prevention and treatment modalities may improve uptake for TGW populations. Also, fear of drug interaction between PrEP and other medicines, particularly female hormones, appeared to be an issue and must be clearly addressed in educational campaigns [40].

Results from a qualitative study to assess health care providers and MSM perspectives on acceptance and feasibility of implementing novel HIV prevention interventions in Brazil showed that although most health care providers were reluctant to engage in new prevention strategies, MSM were very interested in exploring new prevention tools [41]. Increasing PrEP knowledge among potential users and health care providers, especially among physicians, is a key step to facilitating PrEP implementation in our setting.

### Demonstration projects in Brazil

The PrEP Brasil study is a demonstration project (clinical trials.gov NCT 01989611, www.prepbrasil.com.br) designed to evaluate the delivery of PrEP for 450 MSM and TGW for one year. It will generate data to facilitate the decision-making process of incorporating PrEP into the SUS. The project, coordinated by Fiocruz, is ongoing at three sites in Rio de Janeiro (Evandro Chagas National Institute of Infectious Diseases-INI/Fiocruz) and São Paulo (University of São Paulo–USP and São Paulo Referral and Training Center). As of May 2015, the study is fully accrued. Final results are expected by April 2016. As part of the PrEP Brazil project, innovative interventions are being tested to assess their ability to support PrEP users with maintaining treatment adherence and continuing with PrEP usage. PrEP adherence is being supported through the use of text message reminders. In addition, drug concentrations will be measured via plasma and dried blood spot specimens. In addition to the Brazilian National AIDS Program, PrEP Brasil has developed key partnerships with the state AIDS programmes of Rio de Janeiro and São Paulo and two non-governmental organizations, Arco-Iris and Pela VIDDA. The project is jointly funded by the MoH, Fiocruz, and federal and state research funding agencies; Gilead Inc. has donated the study drug (Truvada).

A second demonstration project is scheduled to start by mid-2015 and will enrol 800 MSM, commercial sex workers and drug users across four cities: São Paulo, Porto Alegre, Ribeirão Preto and Fortaleza.

Of note, Truvada for prevention use is not yet approved in Brazil but an application has been filed and is under evaluation by the Brazilian Drug Regulatory Authority.

### Will PrEP be cost-effective in Brazil?

Modelling studies suggest that PrEP can be a cost-effective HIV prevention intervention in developed and developing countries if targeted at individuals at highest risk [37–40]. In Peru, Gomez *et al*. found that cost per DALY averted, assuming the iPrEx profile of adherence (a uniform strategy at a 20% coverage level), ranged from US$1,036 to US$4,254 when considering uncertainty due to PrEP conditional efficacy, which is below the WHO Choosing Interventions That Are Cost-Effective (WHO-CHOICE) threshold for a cost-effective intervention for Peru (2010 per capita GDP of US$5401/DALY) [42]. The WHO-CHOICE considers an intervention to be very cost-effective if its cost is less than the GDP per capita per DALY averted and cost-effective if it costs between one and three times the GDP per capita [41].

Although a PrEP cost-effectiveness model has not yet been developed for Brazil, it is very likely that PrEP would be cost-effective if we consider that the Brazilian and the Peruvian epidemics resemble each other (both have concentrated epidemics with MSM and TGW being most affected), that the model developed by Gomez *et al*. reflects the transmission dynamics between these groups, that the costs and effectiveness of PrEP are similar in Brazil as those estimated for Peru and that Brazil's 2010 per capita GDP was twice that of Peru (US$10,978).

We argue that assuming similar costs and effectiveness in Peru and Brazil is plausible for two reasons. First, regarding PrEP's cost, as the sole procurement agent for ARVs within Brazil, the Brazilian MoH has vast experience in negotiating reasonable pricing strategies from pharmaceutical companies [43] and will likely be able to obtain Truvada at a fair cost that would fit well within the ranges assumed in the Peruvian study. Second, regarding PrEP's effectiveness, the better adherence to Truvada in iPrEX and iPrEX OLE studies observed in the Brazilian sites in comparison with the Lima sites suggests that improved or at least similar levels of effectiveness may be reached in Brazil; this supports cost-effectiveness of PrEP with Truvada in Brazil [43, 44].

The ongoing PrEP demonstration projects and national respondent-driven sampling studies among MSM and TGW will contribute with data to develop a model for PrEP cost-effectiveness analysis that reflects the scenarios in Brazil.

## Conclusions

Since the 1990s, the Brazilian MoH has pushed the envelope with its innovative strategies for HIV prevention, care, treatment and respect for human rights. The success of the Brazilian approach helped demonstrate that universal access to cART is not only an effective treatment strategy but also an efficacious prevention tool [28, 31].

The implementation of PrEP in Brazil is feasible. A synergistic rollout of treatment as prevention and PrEP will maximize public health and individual benefits of the country's comprehensive response to the AIDS epidemic. Intensification of combination prevention strategies at critical points in the HIV transmission cycle is key to achieving the 90–90–90 UNAIDS/WHO targets by 2020 and successfully ending the HIV epidemic in Brazil [45].
